# The Association Between Genomic Heterozygosity and Carcass Merit in Cattle

**DOI:** 10.3389/fgene.2022.789270

**Published:** 2022-02-24

**Authors:** David Kenny, Tara R. Carthy, Craig P. Murphy, Roy D. Sleator, Ross D. Evans, Donagh P. Berry

**Affiliations:** ^1^ Animal and Grassland Research and Innovation Centre, Teagasc, Fermoy, Ireland; ^2^ Department of Biological Sciences, Munster Technological University, Cork, Ireland; ^3^ Animal and Grassland Research and Innovation Centre, Teagasc Grange, Dunsany, Ireland; ^4^ Irish Cattle Breeding Federation, Bandon, Ireland

**Keywords:** association analysis, carcass traits, dominance, genomic heterozygosity, heterosis, non-additive

## Abstract

The objective of the present study was to quantify the association between both pedigree and genome-based measures of global heterozygosity and carcass traits, and to identify single nucleotide polymorphisms (**SNPs**) exhibiting non-additive associations with these traits. The carcass traits of interest were carcass weight (**CW**), carcass conformation (**CC**) and carcass fat (**CF**). To define the genome-based measures of heterozygosity, and to quantify the non-additive associations between SNPs and the carcass traits, imputed, high-density genotype data, comprising of 619,158 SNPs, from 27,213 cattle were used. The correlations between the pedigree-based heterosis coefficient and the three defined genomic measures of heterozygosity ranged from 0.18 to 0.76. The associations between the different measures of heterozygosity and the carcass traits were biologically small, with positive associations for CW and CC, and negative associations for CF. Furthermore, even after accounting for the pedigree-based heterosis coefficient of an animal, part of the remaining variability in some of the carcass traits could be captured by a genomic heterozygosity measure. This signifies that the inclusion of both a heterosis coefficient based on pedigree information and a genome-based measure of heterozygosity could be beneficial to limiting bias in predicting additive genetic merit. Finally, one SNP located on *Bos taurus* (**BTA**) chromosome number 5 demonstrated a non-additive association with CW. Furthermore, 182 SNPs (180 SNPs on BTA 2 and two SNPs on BTA 21) demonstrated a non-additive association with CC, while 231 SNPs located on BTA 2, 5, 11, 13, 14, 18, 19 and 21 demonstrated a non-additive association with CF. Results demonstrate that heterozygosity both at a global level and at the level of individual loci contribute little to the variability in carcass merit.

## Introduction

It is well documented that crossbred cattle exhibit superior performance for a variety of traits when compared to their purebred contemporaries (Gregory et al., 1994; Sørensen et al., 2008; Buckley et al., 2014). Crossbreeding strategies exploit what is referred to as heterosis (Shull, 1914). At the molecular level, heterosis is thought to be due to two possible mechanisms, namely dominance and overdominance (Li et al., 2008). Dominance attributes heterosis to the cancelling, across multiple loci, of inferior recessive alleles inherited from one parent by the superior dominant allele, inherited from the other parent (Davenport, 1908). Overdominance, on the other hand, attributes heterosis to the superior (or inferior) performance of animals heterozygous, at a given locus, compared to the performance of animals homozygous at that same locus (Shull, 1908).

Adjustment for heterosis effects is common practice in the analysis and genetic evaluation of crossbred cattle populations (VanRaden and Sanders, 2003; Williams et al., 2010; Kenny et al., 2020a). To date, this adjustment usually involves the inclusion of a pairwise breed heterosis coefficient (Dickerson, 1973; VanRaden and Sanders, 2003) in the statistical model (VanRaden and Sanders, 2003; Berry et al., 2019; Twomey et al., 2020). Regression coefficients from the regression of animal performance on such breed composition-based heterosis coefficients have been widely reported for a variety of traits in cattle, including carcass traits (i.e., the traits of interest to the present study) (Berry et al., 2018; Wetlesen et al., 2020; Kenny et al., 2021). The use of a heterosis coefficient based on breed composition acts as a proxy for the expected heterozygosity in an animal’s genome (Dickerson, 1973). Nonetheless, with the availability of genotypes from high-density (**HD**) genotyping panels (Hunkapiller et al., 1991), the possibility to directly estimate the level of heterozygosity in an individual’s genome is now feasible (Engelsma et al., 2012; Akanno et al., 2017). In the present study, various genomic heterozygosity measures were defined using imputed, HD genotypes. The objective of the present study was to quantify the relationship between a pedigree-based heterosis coefficient and various genomic heterozygosity measures. Additionally, of interest was the association between the genomic heterozygosity measures and three carcass metrics, namely carcass weight (**CW**), carcass conformation (**CC**) and carcass fat (**CF**). Akanno et al. (2017) previously quantified the relationship between a pedigree-based heterosis coefficient and a genomic heterozygosity measure. These authors also documented associations between a genomic heterozygosity measure and carcass metrics based on the USDA carcass grading system. While Akanno et al. (2017) used a dataset of 1,124 crossbreed cattle, whose breed composition comprised only three breeds (i.e., Angus, Charolais and Hereford), the present study used an edited dataset of 27,213 animals that were crossbred combinations of 12 distinct breeds; these breeds include Angus, Aubrac, Belgium Blue, Blonde d’Aquitaine, Charolais, Hereford, Holstein-Friesian, Jersey, Limousin, Saler, Shorthorn, and Simmental. In addition, the present study considered additional genomic heterozygosity measures to that described by Akanno et al. (2017).

Access to genotype information in livestock has also enabled genome-wide association studies to investigate the contribution of single nucleotide polymorphisms (**SNPs**) to livestock performance; carcass traits are one such suite of traits previously investigated in cattle (McClure et al., 2010; Saatchi et al., 2014; Purfield et al., 2019). While the focus of such studies has primarily been on the additive contribution of SNP alleles, in recent years, the focus has changed to investigate the non-additive contribution of SNP genotypes. Indeed, consideration of such non-additive effects has, in some studies, been reported to improve the accuracy of partitioning the phenotypic variance into its additive and non-additive components, contributing to improvements in genomic evaluations (Su et al., 2012; Aliloo et al., 2016; Moghaddar and Van der Werf, 2017). The association between additive SNP effects and the carcass traits of interest to the present study have been reported previously in cattle (Purfield et al., 2019). On the other hand, few studies have, to the best of our knowledge, yet investigated the existence of non-additive SNP effects for carcass traits (Akanno et al., 2018), with no studies previously using (imputed) high-density SNP data to detect the presence of such effects for carcass traits. Therefore, a further objective of the present study was to conduct an association study to identify SNPs exhibiting non-additive (i.e., dominance) associations with the carcass traits of interest using imputed, high-density genotype data. Results from the present study should provide further insight into the genetic architecture of the carcass traits of interest. Furthermore, should SNPs with significant dominance effects be detected for the carcass traits, this information could potentially be used to improve the accuracy of genomic evaluations for the traits, as well as to inform mate selection programs.

## Materials and Methods

### Genomic Data

Previously imputed HD genotypes, comprising 734,159 autosomal SNPs, were available from a pre-existing database for 638,662 cattle. Prior to imputation, all 638,662 animals had genotype information from a variety of genotyping panels; including the Illumina HD (777,962 SNPs), the Illumina Bovine SNP50 (54,001 SNPs), or one of the custom Irish Dairy and Beef (IDB) genotype panels, namely IDBV1 (16,662 SNPs), IDBV2 (16,223 SNPs) or IDBV3 (52,445 SNPs). The imputation to HD pipeline has been extensively described by Purfield et al. (2019) and was conducted for all genotyped animals using a two-step approach in FImpute2 (Sargolzaei et al., 2014). Only autosomal SNPs with a call rate ≥90% that resided on a known chromosome, and at a known position on UMD 3.1, were considered in the imputation process. In addition, all animals had a call rate ≥90%. The first step involved imputing animals genotyped on the IDB genotype panels to the Illumina Bovine SNP50 density. These animals, along with the animals genotyped on the Illumina Bovine SNP50 panel, were then imputed to HD using a multi-breed reference population of 5,504 HD genotyped animals.

### Phenotypic Data

The three carcass phenotypes of interest in the present study were CW, CC, and CF, measured in accordance with the EUROP grading system. Carcass weight is measured in kg, on average, 1 hour after slaughter, following the removal of the head, hide, legs, thoracic and abdominal organs, and internal fat. With regard to CC and CF, the former reflects the shape and development of the carcass, particularly on the round, back and shoulders, while the latter reflects the level of fat covering the carcass, as well as within the thoracic cavity of the carcass (Kenny et al., 2020b). Under the EUROP grading system, CC scores are represented by the letters E, U, R, O and P (on a scale from best to worst), each of which are subdivided into three subscores (i.e., −, = and +). Carcass fat scores, on the other hand, are represented by the numbers 1 (lowest fact cover), 2, 3, 4 and 5 (highest fat cover), which are also subdivided using the three subscores (i.e., −, = and +). For the purpose of the present study, both CC and CF scores were converted to 15-point numeric scales, with 1 representing the worst conformation and lowest fat cover, and 15 representing the best conformation and highest fat cover.

Only genotyped young bulls, heifers and steers slaughtered between the ages of 12 and 36 months with recorded carcass phenotypes were considered. Based on this criterion, data were available for 93,467 animals. Additionally, any of the young bulls, heifers or steers born to dams with a parity number >10, or from embryo transfer, were removed. Finally, any cattle with more than three inter-herd movements during their life, or a movement to another herd 100 days before slaughter, were not considered further. The birth herd of all remaining animals were categorized as either beef or dairy, based on parameters outlined by Ring et al. (2018). Herds were classified as beef when the average dam breed composition within the herd consisted of ≤65% dairy breeds (i.e., Holstein-Friesian or Jersey), whereas herds with an average dam breed composition consisting of >75% dairy breeds were classified as dairy. All animals born in herds that remained unclassified were removed from the dataset. After the above edits, data remained for 73,040 animals. All remaining genotyped animals with carcass phenotypes were allocated to contemporary groups of finishing herd, year, season and sex using an algorithm that is routinely used in the Irish genetic evaluations (Pabiou et al., 2012). The contemporary groups comprised animals of the same sex that were slaughtered from the same herd within 60 days of one another. All animals in contemporary groups containing less than five animals were removed. After all edits, 27,213 genotyped animals remained, for which the sire and dam of all, along with their carcass phenotypes, were known. In terms of quality control for the genotype data of the 27,213 animals of interest, all SNPs with a minor allele frequency ≤0.05 were removed. After quality control, a total of 619,158 autosomal SNPs remained for the animals of interest, all of which had a known position on UMD 3.1 and were located on known chromosomes. The position of each SNP was subsequently converted to the newest reference *Bos tarsus* genome assemble, namely ARS-UCD 1.2, using the NCBI genome remapping service (https://www.ncbi.nlm.nih.gov/genome/tools/remap).

A general heterosis and recombination loss coefficient was calculated for all animals using the formulae outlined by VanRaden and Sanders (2003):

$$heterosis=1-\;\sum_{i=1}^{n}B\_S_{i}\times \;B\_D_{i}\;$$ (1)

$$recombination\;loss=1-\sum_{i=1}^{n}\frac{B\_D_{i}^{2}+B\_D_{i}^{2}}{2}$$ (2)

where *B_S*_*i*_ and *B_D*_*i*_ are the proportion of breed *i* in the breed composition of the sire and dam, respectively, based on pedigree information recorded in the Irish cattle database. The recombination loss coefficients were categorized as 0%, >0 and ≤10%, >10% and ≤20%, >20% and ≤30%, >30% and ≤40%, >40% and ≤50%, and >50%.

### Genomic Heterozygosity Measures

Three measures of genomic heterozygosity were defined for each animal based on its genotypes, namely observed heterozygosity (**OH**), homozygosity by locus (**HL**) and runs of heterozygosity (**ROHet**). Observed heterozygosity was quantified separately for each animal as:

$$Observed\;heterozygosity=\;\frac{Number\;of\;called\;heterozygous\;SNPs\;}{Total\;number\;of\;called\;SNPs}$$ (3)

Homozygosity by locus (Aparicio et al., 2006) was calculated for each animal using the following formula:

$$Homozygosity\;by\;locus=\;\frac{\sum E_{h}}{\sum E_{h}+\;\sum E_{j}}$$ (4)

where $E_{h}$ is the expected heterozygosity for the loci at which the animal in question was homozygous and $E_{j}$ is the expected heterozygosity for the loci at which the animal is heterozygous. Expected heterozygosity was calculated separately for each locus based on the genotype data of all 27,213 animals as $\;1\;-\;\sum_{i=1}^{n}q_{i}^{2}$ , where $q_{i}$ is the frequency of the $i^{th}$ allele at a given locus.

Finally, ROHet were calculated for each animal using a 50-SNP sliding window in one SNP intervals and the detectRUNS package in R (Biscarini et al., 2019). To define a single ROHet, the run had to have a minimum length of 1 Kb and a minimum density of one SNP every 50 bp. In addition, no more than two missing SNPs and one homozygous SNPs were permitted within the 50-SNP sliding window used to calculate ROHet.

### Statistical Analyses

As per the Kolmogorov-Smirnov test for normality, the pedigree-based heterosis coefficient and the genomic heterozygosity measures were not normally distributed (*p* < 0.05). On that basis, Spearman rank correlation coefficients were calculated between the pedigree-based heterosis coefficient and the three genomic heterozygosity measures (i.e., OH, HL and ROHet).

The association between the pedigree-based heterosis coefficient and the carcass traits, as well as between the genomic heterozygosity measures and the carcass traits, were quantified separately in ASReml 4.2 (Gilmour et al., 2015) using the following linear mixed model:

$$Y_{ijklmn}=par_{j}+birth_{k}+twin_{l}+\;rec\_cla_{m}+cg_{n}+het\;+a_{i\;}+e_{ijklmn}$$ (5)

where $Y_{ijklmn}$ was the recorded carcass phenotype of animal i; $par_{j}$ was the fixed effect of dam parity j ( j = 1, 2, 3, 4 and 5+); $birth_{k}$ was the fixed effect of birth herd type k ( k = 0 (beef) or 1 (dairy)); $\;twin_{l}$ was the fixed effect for whether animal i was born a singleton ( l = 0) or a twin ( l = 1); $rec\_cla_{m}$ was the fixed effect of recombination loss class m ( m = 0–6); $cg_{n}$ was the fixed effect of contemporary group n ; het was the covariate term representing either the pedigree-based heterosis coefficient or one of the genomic heterozygosity measures of animal i ; $a_{i}$ was the random direct genetic effect of animal i and $e_{ijklmn}$ was the random residual effect. The distribution of the random genetic effect was assumed as $\;a\;\sim \;N\left(\mathrm{Qg},A\sigma_{a}^{2}\right)$ , where Q is a matrix relating a with genetic groups, g is a vector of genetic group means, and A and $\sigma_{a}^{2}$ are, respectively, a numerator relationship matrix and the genetic variance. The numerator relationship matrix was constructed by tracing the pedigree of each animal back to their founder animals, who were allocated to the genetic groups represented in the Q matrix. The inclusion of the Q matrix in the numerator relationship, which makes the expectations of the random genetic effect specific to each genetic groups, was achieved by included phantom parents for founder animals in the pedigree file used to create the numerator relationship matrix; the allocation of phantom parents to the founder animals were based on their breed. The distribution of the random residual effect was assumed as $\;e\;\sim \;N\left(0,I\sigma_{e}^{2}\right)$ , where I and $\sigma_{e}^{2}$ are, respectively, the identity matrix and the residual variance.

In a supplementary series of analyses, both the pedigree-based heterosis coefficient and one of the genomic heterozygosity measures were simultaneously included in the statistical model; this is as opposed to the initial analysis, in which the pedigree-based heterosis coefficient and the genomic-based heterozygosity measures were included in separate statistical models. Finally, further analysis was conducted in which the pedigree-based heterosis coefficient, as well as HL and ROHet, or OH and ROHet, were all simultaneously included in the statistical model. Based on variable inflation factors, all of which were <5, the assumption of multicollinearity was not violated when the pedigree-based heterosis coefficient and either one or two of the genomic-based heterozygosity measures, except both HL and OH, were simultaneously included in the analysis. On the other hand, when all three genomic heterozygosity measures were included in the statistical model alongside the heterosis coefficient, the assumption of multicollinearity was violated.

### Genome-Wide Association Analyses

The carcass phenotypes were firstly adjusted for nuisance variables, fitted as fixed effects, as well as for the direct polygenic effect of the animals *via* a genomic relationship matrix fitted as a random effect. The phenotypes were adjusted in the GenABEL package in R (Aulchenko et al., 2007) using the model:

y=1μ+Xβ+Zα+e (6)

where y  was the vector of phenotypes for CW, CC or CF; 1 was a vector of ones; μ was the mean; β was a vector of fixed effects that included contemporary group, birth herd type (i.e., beef or dairy), dam parity and whether the animal was born a singleton or twin; α was a vector of polygenic effects;  e was a vector of random residual effects, and X and Z were incidence matrices for the fixed and random effects, respectively. The distribution of the random effects in the model were assumed as $\;a\;\sim \;N\left(0,G\sigma_{a}^{2}\right)$ , where G and $\sigma_{a}^{2}$ are, respectively, the genomic relationship matrix and the genetic variance, and $\;e\;\sim \;N\left(0,I\sigma_{e}^{2}\right)$ , where I and $\sigma_{e}^{2}$ are the identity matrix and residual variance, respectively. The genomic relationship matrix was constructed using Method I outlined by VanRaden (2008).

Following the adjustment of the phenotypes, a series of association analyses were performed for each locus separately using the model:

$$e=1\mu +b_{1}\alpha_{k}+b_{2}d_{k}+e_{k}$$ (7)

where e is the vector of residuals from model 6;  1 was a vector of ones; μ was the population mean; $\alpha_{k}$ was the vector of additive genotype codes (i.e., AA = 0; AB = 1; BB = 2) for locus  k fitted as a covariate; $d_{k}$ was the vector of dominance genotype codes (i.e., homozygous genotypes = 0; heterozygous genotype = 1) for locus  k fitted as a covariate; $b_{1}$ and $b_{2}$ was the regression coefficient associated with the additive and dominance effects, respectively, and $e_{k}$ was the residuals from this model. Of interest here was the significance of the difference of both $\;b_{1}$ , but, in particular, $b_{2}$ from zero. To correct for multiple testing, all *p* values for $b_{1}$ and $b_{2}$ were separately transformed into q values (Storey and Tibshirani, 2003). Assuming a 1% false-discovery rate (FDR), SNPs with a q value ≤0.01 were considered significant.

### Quantitative Trait Loci Regions

Quantitative trait loci (QTL) regions associated with CW, CC and CF were defined based on the flanking linkage disequilibrium (LD) patterns around the SNPs displaying significant associations. To estimate the start and end positions of the QTL regions, all SNPs within a 0.5 Mb window that were in LD (r^2^ of ≥0.5) with significantly associated SNPs on the same chromosome were considered to be part of a single QTL region. In the case that QTL regions overlapped, these regions were merged together and considered to be a single QTL region. Additionally, the presence of candidate genes within 0.5 Mb of the lead SNPs of the different QTL regions, as well as genes located within the QTL regions, were investigated using ENSEMBL (https://www.ensembl.org/) on the ARS-UCD 1.2 genome assembly, alongside the biomaRt package in R (Durinck et al., 2009).

## Results

### Relationship Between Heterosis and Heterozygosity Measures

Descriptive statistics for the pedigree-based heterosis coefficient and the genomic heterozygosity measures are presented in Supplementary Table S1. The spearman correlation coefficient between the heterosis coefficient, calculated from ancestry information, and OH was 0.76, while the correlation between the heterosis coefficient and HL and between the heterosis coefficient and ROHet was 0.76 and 0.18, respectively (Table 1). In addition, the correlation coefficients between OH and HL, between OH and ROHet, and between HL and ROHet were 0.98, 0.30 and 0.28, respectively.

**TABLE 1:** Spearman correlation coefficients between the pedigree-based heterosis coefficient and the three genomic heterozygosity measures, namely observed heterozygosity, homozygosity by locus and runs of heterozygosity.

	Observed heterozygosity	Homozygosity by locus	Runs of heterozygosity
Heterosis coefficient	0.76	0.76	0.18
Observed heterozygosity	—	0.98	0.30
Homozygosity by locus	—	—	0.28

### Association Between Heterosis and Carcass Merit, and Between Genomic Heterozygosity and Carcass Merit

The association between CW, CC and CF and each of the genomic measures of heterozygosity, along with the associations between the carcass traits and the pedigree-based heterosis coefficient are shown in Table 2. While the range of the heterosis coefficient is bounded between 0 and 1, the range of the genome-based heterozygosity measures is not (Supplementary Table S1). Therefore, for comparative purposes, the values reported are the regression coefficients representing the association between a unit increase in the heterosis coefficient, OH, HL or ROHet and the different carcass traits (Table 2), multiplied by the respective standard deviation of the measure (Supplementary Table S1). For instance, an increase in the standard deviation of the heterosis coefficient, OH, HL and ROHet would be expected to translate into a respective increase of 0.95 kg, 2.10 kg, 2.26 kg and 0.02 kg in CW. For CC, an increase in the standard deviation of the heterosis coefficient, OH, HL and ROHet would be expected to result in a reduction in CC score of −0.07, −0.10, −0.06 and −0.03 units, respectively. Finally, an increase in the standard deviation of the heterosis coefficient, OH, HL and ROHet would be expected to result in a respective increase in CF score of 0.21, 0.38, 0.34 and 0.08 units.

**TABLE 2:** Regression coefficients (standard error in parenthesis) from the regression of carcass weight, carcass conformation and carcass fat on the heterosis coefficient (Het) or one of the genomic heterozygosity measures, namely observed heterozygosity (OH), homozygosity by locus (HL) and runs of heterozygosity (ROHet), and a combination of the heterosis coefficient and one, or more, of the genomic heterozygosity measures simultaneously included in the statistical model.

Heterosis/Heterozygosity measure(s) included in the model	Het^a^	OH^b^	HL^b^	ROHet^c^
Carcass weight (kg)	—	—	—	—
Separate inclusion of each measure	2.78 (0.71)^d^	1.05 (0.16)^d^	1.13 (0.15)^d^	0.003 (0.03)
Het and OH	0.51 (0.82)	0.99 (0.18)^d^	—	—
Het and HL	−0.01 (0.83)	—	1.13 (0.17)^d^	—
Het and ROHet	2.80 (0.71)^d^	—	—	−0.01 (0.03)
Het, OH and ROHet	0.47 (0.83)	1.05 (0.19)^d^	—	−0.05 (0.03)
Het, HL and ROHet	−0.05 (0.83)	—	1.19 (0.18)^d^	−0.06 (0.03)
Carcass conformation [scored 1 (poor) to15 (excellent)]	—	—	—	—
Separate inclusion of each measure	−0.20 (0.03)^d^	−0.05 (0.01)^d^	−0.03 (0.01)^d^	−0.005 (0.001)^d^
Het and OH	−0.13 (0.03)^d^	0.03 (0.001)^d^	—	—
Het and HL	−0.16 (0.03)^d^	—	-0.02 (0.006)^d^	—
Het and ROHet	−0.20 (0.03)^d^	—	—	0.004 (0.001)^d^
Het, OH and ROHet	−0.13 (0.03)^d^	-0.03 (0.01)^d^	—	0.003 (0.001)^d^
Het, HL and ROHet	−0.17 (0.03)^d^	—	-0.01 (0.01)	0.004 (0.001)^d^
Carcass fat [scored 1 (thin) to 15 (fat)]	—	—	—	—
Separate inclusion of each measure	0.62 (0.04)^d^	0.19 (0.01)^d^	0.17 (0.01)^d^	0.013 (0.001)^d^
Het and OH	0.24 (0.05)^d^	0.16 (0.01)^d^	—	—
Het and HL	0.28 (0.05)^d^	—	0.14 (0.01)^d^	—
Het and ROHet	0.60 (0.04)^d^	—	—	0.01 (0.002)^d^
Het, OH and ROHet	0.24 (0.05)^d^	0.16 (0.01)^d^	—	0.003 (0.002)
Het, HL and ROHet	0.29 (0.05)^d^	—	0.13 (0.01)^d^	0.004 (0.002)^d^

a Represents an increase of 1 in the heterosis coefficient.

b Represents an increase of 0.01 in observed heterozygosity or homozygosity by locus.

c Represents an increase of 1 in runs of heterozygosity.

d Represent a significant association between the carcass trait and the measure in question (*p* ≤ 0.05).

With the exception of when CW was the dependent variable, both the heterosis coefficient and OH was statistically significant (*p* ≤ 0.05) when included together in the model (Table 2). Similarly, with the exception of when CW was the dependent variable, when the heterosis coefficient and HL, or the heterosis coefficient and ROHet, were simultaneously included in the statistical model both terms were also significant in the model (*p* ≤ 0.05) (Table 2). Finally, in no case was the heterosis coefficient and two genomic heterozygosity measures (i.e., ROH, and HL or OH) all independently associated with the dependent variable; the exception was when CC was the dependent variable and the heterosis coefficient, OH and ROHet were included in the model (Table 2). Furthermore, this was also the case when CF was the dependent variable, and the heterosis coefficient, HL and ROHet were included as independent variables (Table 2). Additionally, the Akaike information criterion values associated with the statistical models including the different combinations of the heterozygosity measures are in Supplementary Table S2. Of the models that included a single measure of heterozygosity as a fixed effect, alongside the nuisance variables, the Akaike information criterion values associated with the models that included a genomic heterozygosity measure, excluding ROHet, were generally lower that those associated with the models that included the heterosis coefficient. When CW was the dependent variable, the lowest Akaike’s information criterion value was associated with the model that included only HL, alongside the other nuisance variables, as fixed effects. When CC and CF were the dependent variables, the lowest Akaike information criterion value was associated with the model that included both the heterosis coefficient and OH as fixed effects, alongside the nuisance variables.

### Genome-wide Association Analyses

**FIGURE 1:**
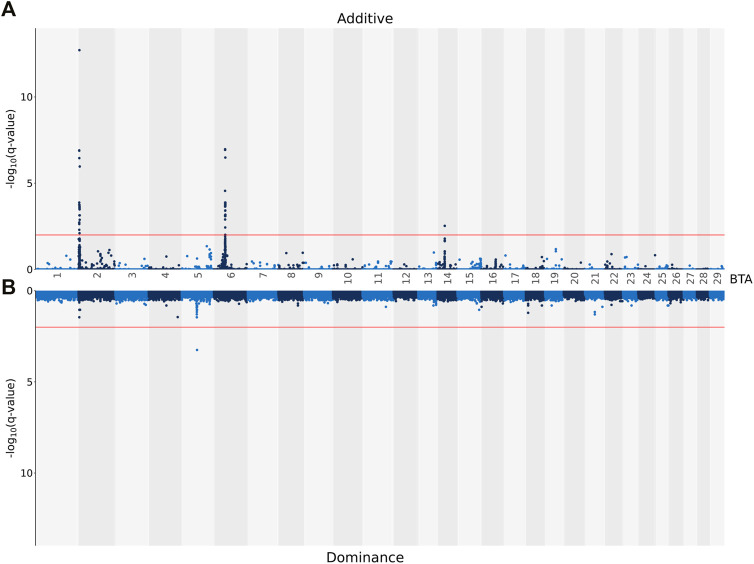
Manhattan plots showing–log_10_ (q-values) of the association between the additive [**(A)** graph] and dominance [**(B)** graph] effect of each single nucleotide polymorphism (SNP) from each *Bos taurus* (BTA) chromosome and the adjusted carcass weights. The red lines represent the threshold for significant (q values ≤0.01) SNPs.

The genomic inflation factor was estimated for all association analyses, and, regardless of the carcass trait under investigation, all were <1.1. This signifies that there was little to no population stratification following the pre-adjustment for the genomic relationship between the animals. The associations, both on an additive and dominance basis, between each SNP and CW are shown in Figure 1. A total of 74 SNPs demonstrated an additive association with CW, while only one SNP (i.e., rs137805316) on *Bos taurus* (**BTA**) chromosome number 5 demonstrating a dominance association with CW. Based on the SNPs demonstrating an additive association with CW, a total of seven distinct QTL regions were identified, four of which were located on BTA 2, with two QTL regions located on BTA 6 and a single QTL region located on BTA 14 also identified (Table 3). The lead SNPs (i.e., the most strongly associated SNP) within the QTL regions defined on BTA 2 and BTA 14 were all intergenic (Table 3). The lead SNPs located in the QTL region on BTA 6 (i.e., rs137720687), stretching from 39.22 to 39.77 Mb, was an intronic variant of SLIT2, while the lead SNP of the other QTL region on BTA 6 (i.e., rs135203216) was an intronic variant of KCNIP4. In addition, the SNP on BTA 5 demonstrating a dominance association with CW was intergenic.

**TABLE 3:** Details of quantitative trait loci (QTL) regions comprising single nucleotide polymorphisms (SNPs) with significant additive associations with carcass weight, namely chromosome number (BTA), start and end position, number of significant SNPs, and details of the most significant SNP of each, namely name, position, effect and frequency (Freq) of the major allele, *p*-value representing the significance difference of the effect from zero, name of nearest gene, SNP annotation, and other genes within 500 Kb of the lead SNP.

BTA	QTL			Most strongly associated SNP in QTL							Other genes in region
	Start	End	SNPs	SNP name	Position	Effect	Freq	*p*-value	Gene	Annotation	
2	2,152,312	2,214,590	2	rs134902036	2,214,590	-1.84	0.72	4.09 × 10^–12^	PLEKHB2	Downstream	ARHGEF4, FAM168B
2	2,329,987	2,333,917	3	rs109052645	2,329,987	-0.826	0.64	4.88 × 10^–7^	PLEKHB2	Downstream	ARHGEF4, FAM168B
2	3,502,283	3,524,560	7	rs109607574	3,510,323	1.132	0.67	1.34 × 10^–10^	—	Intergenic	—
2	4,099,666	4,101,545	3	rs135686370	4,100,735	1.132	0.85	3.16 × 10^–10^	HS6ST1	Upstream	UGGT1, SAP130
6	39,221,671	39,769,206	16	rs137720687	39,425,007	1.276	0.88	3.29 × 10^–15^	SLIT2	Intron	—
6	40,170,015	40,326,680	16	rs135203216	40,238,070	1.215	0.95	3.58 × 10^–13^	KCNIP4	Intron	SLIT2, PACRGL
14	26,969,707	26,971,048	2	rs132861240	26,969,707	1.241	0.78	3.31 × 10^–7^	ASPH	Downstream	CHD7, CLVS1

**FIGURE 2:**
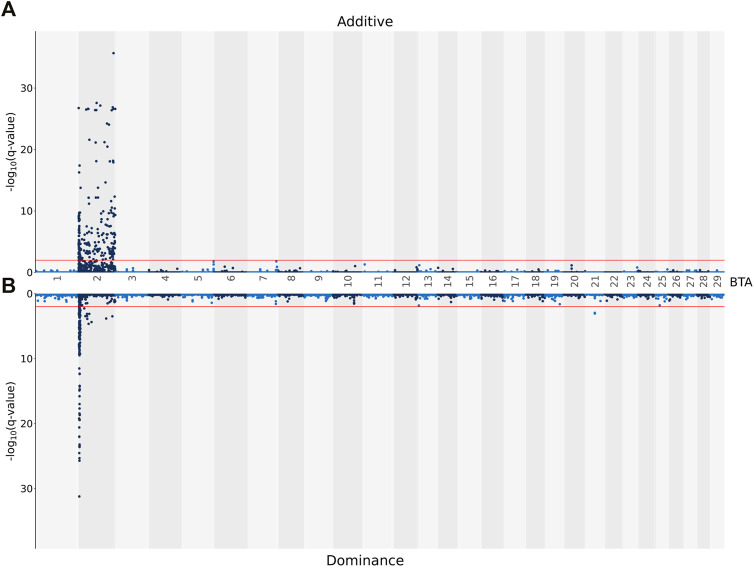
Manhattan plots showing–log10 (q-values) of the association between the additive [**(A)** graph] and dominance [**(B)** graph] effect of each single nucleotide polymorphism (SNP) from each Bos taurus (BTA) chromosome and the adjusted carcass conformation scores. The red lines represent the threshold for significant (q values ≤0.01) SNPs.

The additive and dominance associations between each of the 619,158 investigated SNPs and CC are in shown Figure 2. Of these, 226 SNPs demonstrated an additive association with CC, while 182 SNPs demonstrated a dominance association with CC. All SNPs demonstrating an additive association with CC were located on BTA 2 and collapsed into 19 distinct QTL regions, ranging from 0.92 to 280.08 Kb in length (Table 4). The lead SNPs of the various additive QTL regions included SNPs located within the genes OCA2 (i.e., rs109043505), HERC2 (i.e., rs109878315), TUBGCP5 (i.e., rs109730024), ARHGEF4 (i.e., rs10967377), HS6ST1 (i.e., rs110759081), LIMS2 (i.e., rs110482569) and BIN1 (i.e., rs134297176) (Table 4). Of the 182 SNPs with significant dominance associations with CC, all were located on BTA 2, except for two SNPs (i.e., rs109386755 and rs133273689) located 6.11 Kb apart on BTA 21. Based on the SNPs with a significant dominance association with CC, 21 QTL regions were defined, ranging from 1.24 to 342.28 Kb in length (Table 5). Of the various lead SNPs within the dominance QTL regions, six of the lead SNPs were intronic variants located within TUBGCP5, ARHGEF4, HS6ST1, BIN1, INPP1 and HIBCH, while all other lead SNPs were intergenic variants.

**TABLE 4:** Details of quantitative trait loci (QTL) regions comprising single nucleotide polymorphisms (SNPs) with significant additive associations with carcass conformation, namely chromosome number (BTA), start and end position, number of significant SNPs, and details of the most significant SNP of each, namely name, position, effect and frequency (Freq) of the major allele, *p*-value representing the significance difference of the effect from zero, name of nearest gene, SNP annotation, and other genes within 500 Kb of the lead SNP.

BTA	QTL			Most strongly associated SNP in QTL							Other genes in region
	Start	End	SNPs	SNP name	Position	Effect	Freq	*p*-value	Gene	Annotation	
2	565,339	569,844	2	rs109043505	565,339	0.047	0.81	1.60 × 10^–14^	OCA2	Intron	LGSN, HERC2, NIPA1, NIPA2
2	682,101	791,879	6	rs109878315	729,681	-0.035	0.87	8.95 × 10^–9^	HERC2	Intron	LGSN, OCA2, NIPA1, CYFIP1
2	1,248,113	1,283,778	5	rs109730024	1,283,778	0.048	0.63	1.96 × 10^–14^	TUBGCP5	Intron	HERC2, NIPA1, NIPA2, CYFIP1, CCDC115, IMP4, PTPN18, AMER3, ARHGEF4
2	1,619,549	1,664,976	15	rs133205474	1,622,661	0.047	0.85	6.16 × 10^–14^	ARHGEF4	Upstream	CYFIP1, TUBGCP5, CCDC115, IMP4, PTPN18, AMER3, FAM168B, PLEKHB2
2	1,846,469	1,848,495	3	rs109673771	1,846,469	0.032	0.76	2.95 × 10^–8^	ARHGEF4	Intron	PTPN18, AMER3, FAM168B, PLEKHB2
2	2,152,312	2,214,590	2	rs134902036	2,214,590	-0.066	0.72	1.77 × 10^–26^	PLEKHB2	Downstream	ARHGEF4, FAM168B
2	2,230,600	2,423,609	16	rs110980261	2,361,337	0.058	0.73	3.01 × 10^–23^	PLEKHB2	Downstream	ARHGEF4,FAM168B
2	2,921,455	2,924,516	2	rs136598546	2,924,516	0.042	0.64	2.31 × 10^–9^	—	Intergenic	—
2	2,994,646	3,014,162	2	rs135773145	3,014,162	-0.085	0.67	3.41 × 10^–42^	—	Intergenic	—
2	3,227,403	3,301,918	13	rs110614558	3,236,835	0.081	0.55	3.45 × 10^–33^	—	Intergenic	—
2	3,433,179	3,713,255	15	rs133762933	3,713,255	0.081	0.54	8.59 × 10^–34^	HS6ST1	Upstream	—
2	3,904,008	3,971,148	15	rs136098660	3,941,047	0.055	0.51	7.20 × 10^–19^	HS6ST1	Upstream	UGGT1
2	4,099,666	4,101,545	3	rs135686370	4,100,735	0.078	0.85	1.50 × 10^–27^	HS6ST1	Upstream	UGGT1, SAP130
2	4,216,110	4,226,751	6	rs110759081	4,216,110	-0.071	0.89	1.45 × 10^–32^	HS6ST1	Intron	UGGT1, SAP130, AMMECR1L, POLR2D, WDR33
2	4,591,262	4,607,827	2	rs42904822	4,591,262	0.053	0.81	4.37 × 10^–9^	AMMECR1L	Upstream	HS6ST1,UGGT1, SAP130, POLR2D, WDR33, SFT2D3, LIMS2, GPR17, MYO7B, IWS1
2	4,860,070	4,861,022	2	rs110482569	4,861,022	0.049	0.66	4.09 × 10^–14^	LIMS2	Intron	UGGT1, SAP130, AMMECR1L, POLR2D, WDR33, SFT2D3, GPR17, MYO7B, IWS1, PROC, MAP3K2, ERRC2, CYP27C1
2	5,109,012	5,128,788	4	rs108948452	5,115,682	-0.045	0.85	2.49 × 10^–13^	PROC	Downstream	AMMECR1L, POLR2D, WDR33, SFT2D3, GPR17, MYO7B, IWS1, PROC, MAP3K2, ERRC2, CYP27C1, BIN1
2	5,374,531	5,480,052	8	rs134297176	5,423,184	0.059	0.53	2.14 × 10^–21^	BIN1	Intron	MYO7B, IWS1, MAP3K2, ERCC3, CYP27C1, NAB1, NEMP2, MFSD6
2	5,931,228	5,937,091	4	rs137651762	5,931,228	0.065	0.68	1.28 × 10^–14^	INPP1	Upstream	BIN1, NAB1, NEMP2, MFSD6, HIBCH, MSTN

**TABLE 5:** Details of quantitative trait loci (QTL) regions comprising single nucleotide polymorphisms (SNPs) with significant dominance associations with carcass conformation, namely chromosome number (BTA), start and end position, number of significant SNPs, and details of the most significant SNP of each, namely name, position, effect (Effect) and frequency (Freq) of the heterozygous genotype, *p*-value representing the significance of the difference of the effect from zero, name of nearest gene, annotation, and other genes within 0.5 Mb of the lead SNP.

BTA	QTL			Most strongly associated SNP in QTL							Other genes in region
	Start	End	SNPs	SNP name	Position	Effect	Freq	*p*-value	Gene	Annotation	
2	1,248,113	1,283,778	3	rs109730024	1,283,778	−0.048	0.47	1.35 × 10^–7^	TUBGCP5	Intron	HERC2, NIPA1, NIPA2, CYFIP1, CCDC115, IMP4, PTPN18, AMER3, ARHGEF4
2	1,574,632	1,579,045	2	rs134698928	1,579,045	−0.061	0.10	1.59 × 10^–12^	ARHGEF4	Upstream	CYFIP1, TUBGCP5, CCDC115, IMP4, PTPN18, AMER3, FAM168B, PLEKHB2
2	1,619,549	1,647,935	10	rs133205474	1,622,661	−0.063	0.49	1.13 × 10^–12^	ARHGEF4	Upstream	CYFIP1, TUBGCP5, CCDC115, IMP4, PTPN18, AMER3, FAM168B, PLEKHB2
2	1,847,259	1,848,495	2	rs109701201	1,848,495	−0.041	0.36	2.34 × 10^–6^	ARHGEF4	Intron	PTPN18, AMER3, FAM168B, PLEKHB2
2	2,152,312	2,214,590	2	rs134902036	2,214,590	−0.067	0.16	3.77 × 10^–14^	PLEKHB2	Downstream	ARHGEF4, FAM168B
2	2,230,600	2,434,950	21	rs110980261	2,361,337	−0.99	0.45	1.92 × 10^–30^	PLEKHB2	Downstream	ARHGEF4, FAM168B
2	2,591,903	2,934,187	13	rs133659602	2,780,706	−0.065	0.44	3.48 × 10^–14^	—	Intergenic	—
2	3,212,823	3,264,352	9	rs110614558	3,236,835	−0.113	0.43	6.48 × 10^–32^	—	Intergenic	—
2	3,433,179	3,735,840	15	rs109107915	3,502,283	−0.105	0.49	2.08 × 10^–29^	—	Intergenic	—
2	3,752,032	3,754,081	2	rs136576511	3,754,081	−0.064	0.33	1.51 × 10^–6^	HS6ST1	Upstream	—
2	3,904,008	3,914,796	3	rs134035605	3,904,008	−0.065	0.44	1.43 × 10^–13^	HS6ST1	Upstream	UGGT1
2	3,938,844	3,971,148	11	rs136098660	3,941,047	−0.083	0.43	8.99 × 10^–20^	HS6ST1	Upstream	UGGT1
2	3,987,043	3,988,311	2	rs135950969	3,987,043	−0.067	0.50	1.80 × 10^–7^	HS6ST1	Upstream	UGGT1, SAP130
2	4,021,245	4,101,545	5	rs135686370	4,100,735	−0.097	0.46	1.09 × 10^–24^	HS6ST1	Upstream	UGGT1, SAP130
2	4,186,409	4,189,376	2	rs132939901	4,186,409	−0.047	0.49	1.63 × 10^–7^	HS6ST1	Upstream	UGGT1, SAP130, AMMECR1L, POLR2D, WDR33
2	4,202,001	4,335,970	18	rs136251990	4,212,079	−0.096	0.38	9.13 × 10^–20^	HS6ST1	Intron	UGGT1, SAP130, AMMECR1L, POLR2D, WDR33
2	4,591,262	4,607,827	2	rs42904822	4,591,262	−0.068	0.20	8.14 × 10^–9^	AMMECR1L	Upstream	HS6ST1, UGGT1, SAP130, AMMECR1L, POLR2D, WDR33, SFT2D3, LIMS2, GPR17, MYO7B, IWS1
2	5,328,432	5,358,183	7	rs109091526	5,358,183	−0.065	0.48	1.34 × 10^–8^	CYP27C1	Downstream	LIMS2, GPR17, MYO7B, IWS1, PROC, MAP3K2, ERCC3, BIN1, NAB1, NEMP2, MFSD6
2	5,370,442	5,423,184	4	rs134297176	5,423,184	−0.059	0.30	4.86 × 10^–11^	BIN1	Intron	MYO7B, IWS1, PROC, MAP3K2, ERCC3, BIN1, NAB1, NEMP2, MFSD6
2	5,931,228	5,937,091	4	rs109266532	5,935,155	0.012	0.47	6.08 × 10^–10^	INPP1	Intron	BIN1, NAB1, NEMP2, MFSD6, HIBCH, MSTN, PMS1
2	6,005,043	6,008,468	2	rs13544915	6,005,043	−0.072	0.15	6.07 × 10^–10^	HIBCH	Intron	NAB1, NEMP2, MFSD6, INPP1, MSTN, PMS1

**FIGURE 3:**
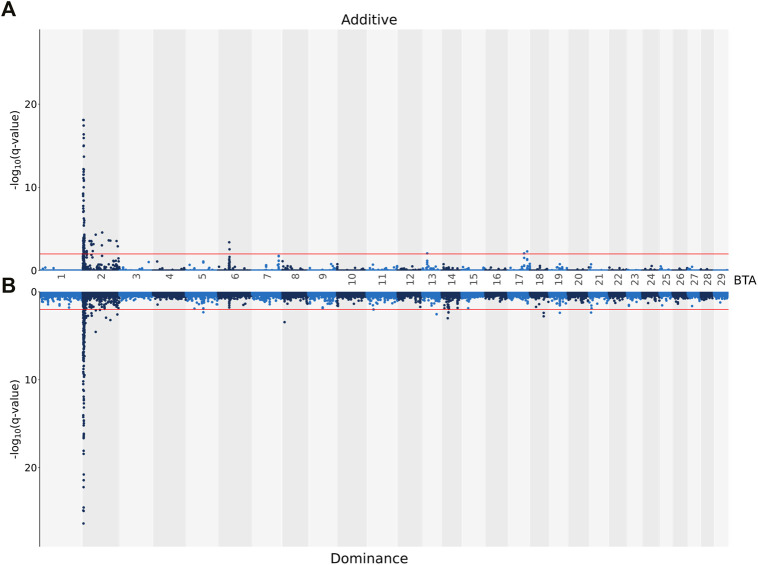
Manhattan plots showing–log_10_ (q-values) of the association between the additive [**(A)** graph] and dominance [**(B)** graph] effect of each single nucleotide polymorphism (SNP) from each *Bos taurus* (BTA) chromosome and the adjusted carcass fat scores. The red lines represent the threshold for significant (q values ≤0.01) SNPs.

In total, 114 SNPs demonstrated an additive association with CF, while 231 SNPs demonstrated a dominance association with CF (Figure 3). With the exception of two SNP located on BTA 6, a SNP on BTA 13 and four SNPs on BTA 17, all SNPs demonstrating additive associations with CF were located on BTA 2 and collapsed into 14 QTL regions that ranged in length from 1.73 to 105.52 Kb. Of the various additive QTL regions defined for CF, the lead SNPs were located within the genes TUBGCP5 (i.e., rs134533754), WRD33 (i.e., rs135023953) and BIN1 (i.e., rs134297176) (Table 6). Of the SNPs demonstrating a dominance association with CF, 212 SNPs were located on BTA 2, which collapsed into 16 different QTL regions. In addition, single SNPs on BTA 5, BTA 19 and BTA 21 demonstrated dominance associations with CF, with a further three SNPs located on BTA 11, two SNPs on BTA 13, four SNPs on BTA 14 and four SNPs on BTA 18 also demonstrating dominance associations with CF. These SNPs collapsed into single QTL regions on BTA 8, BTA 11, BTA 13, BTA 14 and BTA 18 (Table 7). The lead SNPs of the different dominance QTL regions included various intergenic variants, as well as intronic variants located in TUBGCP5, HS6ST1, HIBCH, THUMPD2 and LSM14A (Table 7).

**TABLE 6:** Details of quantitative trait loci (QTL) regions comprising single nucleotide polymorphisms (SNPs) with significant additive associations with carcass fat, namely chromosome number (BTA), start and end position, number of significant SNPs, and details of the most significant SNP of each, namely name, position, effect and frequency (Freq) of the major allele, *p*-value representing the significance difference of the effect from zero, name of nearest gene, SNP annotation, and other genes within 0.5 Mb of the lead SNP.

BTA	QTL			Most strongly associated SNP in QTL							Other genes in region
	Start	End	SNPs	SNP name	Position	Effect	Freq	*p*-value	Gene	Annotation	
2	1,248,113	1,283,778	5	rs134533754	1,248,113	−0.070	0.67	2.61 × 10^–14^	TUBGCP5	Intron	HERC2, NIPA1, NIPA2, CYFIP1, CCDC115, IMP4, PTPN18, AMER3, ARHGEF4
2	1,619,549	1,664,976	9	rs133205474	1,622,661	−0.064	0.85	8.20 × 10^–12^	ARHGEF4	Upstream	CYFIP1, TUBGCP5, CCDC115, IMP4, PTPN18, AMER3, FAM168B, PLEKHB2
2	2,152,312	2,214,590	2	rs134902036	2,214,590	0.080	0.72	1.92 × 10^–9^	PLEKHB2	Downstream	ARHGEF4, FAM168B
2	2,329,987	2,336,742	4	rs110108312	2,333,917	0.049	0.65	8.52 × 10^–8^	PLEKHB2	Downstream	ARHGEF4, FAM168B
2	2,361,337	2,401,930	2	rs110957350	2,401,930	0.077	0.73	3.55 × 10^–18^	PLEKHB2	Downstream	FAM168B
2	2,994,646	3,014,162	2	rs135773145	3,014,162	0.091	0.67	7.04 × 10^–22^	—	Intergenic	—
2	3,227,403	3,264,352	5	rs109033262	3,227,403	-0.094	0.72	2.48 × 10^–20^	—	Intergenic	—
2	3,502,283	3,526,094	9	rs109354447	3,514,143	−0.087	0.79	5.07 × 10^–19^	—	Intergenic	—
2	3,909,497	3,914,796	2	rs109822566	3,914,796	−0.049	0.60	1.36 × 10^–7^	HS6ST1	Upstream	UGGT1
2	3,938,844	3,960,478	6	rs136098660	3,941,047	−0.057	0.51	1.77 × 10^–9^	HS6ST1	Upstream	UGGT1, SAP130
2	4,099,666	4,101,545	3	rs132696854	4,101,545	−0.076	0.85	2.38 × 10^–14^	HS6ST1	Upstream	UGGT1, SAP130, AMMECR1L
2	4,216,110	4,217,841	2	rs110759081	4,216,110	0.081	0.88	2.27 × 10^–19^	HS6ST1	Intron	UGGT1, SAP130, AMMECR1L, POLR2D, WDR33
2	4,752,309	4,784,432	2	rs135023953	4,752,309	0.072	0.88	1.68 × 10^–16^	WDR33	Intron	UGGT1, SAP130, AMMECR1L, POLR2D,SFT2D3, LIMS2, GPR17, MYO7B, IWS1, PROC, MAP3K2, ERCC3
2	5,374,531	5,480,052	5	rs134297176	5,423,184	-0.059	0.53	2.77 × 10^–10^	BIN1	Intron	MYO7B, IWS1, PROC, MAP3K2, ERCC3, CYP27C1, NAB1, NEMP2, MFSD6

**TABLE 7:** Details of quantitative trait loci (QTL) regions comprising single nucleotide polymorphisms (SNPs) with significant dominance associations with carcass fat, namely chromosome number (BTA), start and end position, number of significant SNPs, and details of the most significant SNP of each, namely name, position, effect (Effect) and frequency (Freq) of the heterozygous genotype, *p*-value representing the significance of the difference of the effect from zero, name of nearest gene, annotation, and other genes within 0.5 Mb of the lead SNP.

BTA	QTL			Most strongly associated SNP in QTL							Other genes in region
	Start	End	SNPs	SNP name	Position	Effect	Freq	*p*-value	Gene	Annotation	
2	1,248,113	1,290,025	5	rs109730024	1,283,778	0.094	0.46	8.27 × 10^–8^	TUBGCP5	Intron	HERC2, NIPA1, NIPA2, CYFIP1, CCDC115, IMP4, PTPN18, AMER3, ARHGEF4
2	1,574,632	1,579,045	2	rs134698928	1,579,045	0.106	0.49	4.37 × 10^–15^	ARHGEF4	Upstream	CYFIP1, TUBGCP5, CCDC115, IMP4, PTPN18, AMER3, FAM168B, PLEKHB2
2	1,619,549	1,664,976	17	rs43284356	1,643,777	0.101	0.46	1.04 × 10^–12^	ARHGEF4	Upstream	CYFIP1, TUBGCP5, CCDC115, IMP4, PTPN18, AMER3, FAM168B, PLEKHB2
2	2,152,312	2,214,590	2	rs134902036	2,214,590	0.095	0.40	2.99 × 10^–12^	PLEKHB2	Downstream	ARHGEF4, FAM168B
2	2,230,600	2,401,930	20	rs110957350	2,401,930	0.140	0.40	7.84 × 10^–33^	PLEKHB2	Downstream	FAM168B
2	2,591,903	2,936,048	13	rs137575666	2,933,071	0.117	0.49	3.78 × 10^–11^	—	Intergenic	—
2	3,205,071	3,301,918	15	rs108984792	3,228,751	0.142	0.43	8.35 × 10^–24^	—	Intergenic	—
2	3,433,179	3,526,094	11	rs109354447	3,514,143	0.151	0.33	6.14 × 10^–27^	—	Intergenic	—
2	3,904,008	3,914,796	3	rs109822566	3,914,796	0.115	0.45	2.51 × 10^–17^	HS6ST1	Upstream	UGGT1
2	3,938,844	3,971,148	9	rs136098660	3,941,047	0.130	0.50	2.39 × 10^–21^	HS6ST1	Upstream	UGGT1
2	4,099,666	4,101,545	3	rs135686370	4,100,735	0.099	0.25	5.43 × 10^–12^	HS6ST1	Upstream	UGGT1, SAP130
2	4,210,568	4,363,340	11	rs110759081	4,216,110	0.088	0.20	1.74 × 10^–11^	HS6ST1	Intron	UGGT1, SAP130, AMMECR1L, POLR2D, WDR33
2	5,057,634	5,087,336	5	rs136843388	5,077,763	0.131	0.45	1.11 × 10^–9^	PROC	Upstream	MYO7B, IWS1, MAP3K2,ERCC3, CYP27C1, BIN21
2	5,349,576	5,358,183	3	rs109091526	5,358,183	0.082	0.47	3.42 × 10^–10^	CYP27C1	Downstream	LIMS2, GPR17, MYO7B, IWS1, PROC, MAP3K2, ERCC3, BIN1, NAB1, NEMP2, MFSD6
2	5,371,014	5,423,184	2	rs109684524	5,371,014	0.089	0.47	7.89 × 10^–11^	BIN1	Upstream	LIMS2, GPR17, MYO7B, IWS1, PROC, MAP3K2, ERCC3, CYP27C1, NAB1, NEMP2, MFSD6
2	6,052,068	6,052,977	2	rs110937765	6,052,977	0.097	0.39	4.44 × 10^–12^	HIBCH	Intron	NAB1, NEMP2, MFSD6, INPP1, MSTN, PMS1
8	8,086,908	8,092,859	3	rs135213669	8,089,690	0.013	0.39	9.45 × 10^–8^	MTMR9	Downstream	FDFT1, NEIL2, GATA4, BLK, FAM167A, TDH, XKR6, PINX1
11	22,056,688	22,059,527	3	rs43673407	22,059,527	0.063	0.50	3.91 × 10^–6^	THUMPD2	Intron	MAP4K3, TMEM178A, SLC8A1
13	59,936,331	59,936,944	2	rs136199851	59,936,944	0.065	0.48	9.15 × 10^–7^	SIRPD	Downstream	FAM209A, RTF2, GCNT7, CASS4, CSTF1, AURKA, FAM210B, MC3R
14	21,713,699	25,937,514	3	rs41725494	25,934,977	0.089	0.49	1.71 × 10^–6^	CA8	Upstream	RAB2A, CHD7
18	44,742,185	44,751,188	3	rs43088759	44,749,396	0.066	0.21	1.39 × 10^–6^	LSM14A	Intron	LSM14A, GPI, PDCD2L, UBA2, WTIP

## Discussion

The majority of livestock research studies that use genomic information focus on the additive association of SNPs with traits of importance (Bolormaa et al., 2011; Twomey et al., 2019; Scholtens et al., 2020). Of course, knowledge of SNP-phenotype additive associations are important for genomic predictions of additive genetic merit, in the pursuit of genetic gain (Meuwissen et al., 2001). In the case of carcass traits, the additive associations between SNPs and carcass performance is well documented in previous cattle studies (McClure et al., 2010; Saatchi et al., 2014; Purfield et al., 2019). Nonetheless, non-additive SNP effects, namely the phenomenon known as heterosis and its underlining molecular mechanism known as dominance, are important considerations, especially in relation to mate selection and the prediction of actual phenotypic performance. The benefits of exploiting heterosis to improve phenotypic performance have been widely reported in previous cattle studies (Berry et al., 2018; Wetlesen et al., 2020). Furthermore, the need to properly adjust for the effects of heterosis in the analysis of crossbred livestock populations is well known (Dickerson, 1973; VanRaden and Sanders, 2003; Williams et al., 2010).

The heterosis coefficient, which acts as proxy for expected heterozygosity (Dickerson, 1973), is calculated based on the breed composition of the animal’s parents (VanRaden and Sanders, 2003) and is primarily used to account for the effects of heterosis in the analysis of crossbred populations (VanRaden and Sanders, 2003; Berry et al., 2019; Twomey et al., 2020). It has been reported that pigs with higher heterosis coefficients (i.e., F1 cross pigs) tend to exhibit greater levels of heterozygosity (Iversen et al., 2019). Alternatively, the use of genomic information to more directly infer genomic heterozygosity has been suggested in previous livestock studies for the adjustment of heterosis effects in crossbred livestock populations (Akanno et al., 2017; Iversen et al., 2019). Compared to the heterosis coefficient, the use of genomic information to define global genomic heterozygosity measures is not compromised by errors in pedigree information and also accounts for segregation during gametogenesis. Nonetheless, it should be noted, an important consideration associated with the use of imputed genotypes to infer genomic heterozygosity is that the genotypes are accurately imputed (Li et al., 2009). Furthermore, knowledge of the regions within the genome that demonstrate (significant) dominance effects for a trait could be used for the definition of trait-specific genomic heterozygosity measures for the traits. The definition of such trait-specific genomic heterozygosity measures, as opposed to the global definitions used in the present study, could be beneficial to the proper adjustment for heterosis effects.

### Heterozygosity Measures and Carcass Merit Associations

No previous study has, to the best of our knowledge, reported correlations between the pedigree-based heterosis coefficient, as defined herein, and genomic-based heterozygosity measures. The Spearman correlations reported between the pedigree-based heterosis coefficient and both HL and OH in the present study signifies that the rankings based on expected heterozygosity inferred from an animal’s breed composition is moderately related to those based on OH and HL. A unity correlation is not, of course, expected. Based on the squared correlation coefficient, 57.8% of the variance in the rankings based on OH and HL can be explained by rankings on the heterosis coefficient. In contrast, given the square of the correlation reported in the present study between the heterosis coefficient and ROHet, just 3.2% of the variance in the rankings based on ROHet is explained by those based on the heterosis coefficient. Compared to HL and OH, which reflect the proportion of called SNPs that were heterozygous with and without weightings based on expected heterozygosity, respectively, ROHet reflects the number of stretches comprising consecutive heterozygous SNP genotypes in an animal’s genome (Williams et al., 2016). Nonetheless, there should be some overlap between these measures, with the SNPs present in a ROHet also used to calculate the extent of OH or HL in an animal’s genome. When OH was recalculated for each animal excluding the SNPs located in that animal’s ROHet, the Spearman rank correlation between this measure of OH and the values of OH calculated using all SNPs was 0.99. Furthermore, when the number of ROHet in an animal’s genome were categorized based on the length of the runs, the strength of the relationship between the number of ROHet and the heterosis coefficient weakened as the category of length of the ROHet increased. The Spearman correlation between the number of short (≤150 Kb) ROHet and the heterosis coefficient was 0.15, while the corresponding correlation for number of intermediate (151 Kb–300 Kb) ROHet and the number of long (>300 Kb) ROHet was 0.13 and 0.09, respectively. Additionally, the correlation of 0.98 between OH and HL in the present study signifies that there was little to no difference in the ranking of animals based on these two metrics. This indicates that, for the genotypes used in the present study, the differentiation of animals based on the extent of heterozygosity in their genomes is similar whether or not weightings based on expected heterozygosity are considered. The associations between the pedigree-based heterosis coefficient in cattle and the carcass traits of interest to the present study have been previously documented in cattle (Berry et al., 2018; Kenny et al., 2021). The positive associations between the heterosis coefficient and both CW and CF reported herein is in agreement with the associations reported in previous cattle studies (Berry et al., 2018; Kenny et al., 2021). Furthermore, Kenny et al. (2021) reported negative associations between an increase in the heterosis coefficient and CC, which is in agreement with the present study, while, on the contrary, Berry et al. (2018) reported a positive association between the heterosis coefficient and CC. Kenny et al. (2021) attributed this discrepancy to the fact that the data used in their study, as well as herein, comprised beef and dairy-origin animals, while those used by Berry et al. (2018) comprised only dairy-origin animals. The regression coefficient from the regression of CW, CC and CF on the heterosis coefficient in the present study was equivalent to 10.6, 19.8 and 66.0% of the corresponding genetic standard deviation, respectively. The genetic standard deviations were 26.16 kg for CW, 1.01 units (scored 1–15) for CC and 0.94 units (scored 1–15) for CF as estimated by Kenny et al. (2020a).

On the other hand, the association between global genomic heterozygosity measures and the carcass traits of interest to the present study have yet to be reported in cattle. This is with the exception of the study regressing CW on OH (Akanno et al., 2017), where no association (*p* > 0.05) between OH and hot CW was detected in the 1,124 cattle in their study. Results from the present study demonstrated that the heterosis coefficient and a genomic-based heterozygosity measure, when simultaneously included in the analysis, were associated (*p* ≤ 0.05) with both CC and CF, but not with CW. This signifies that, despite the fact that these measures all relate to heterozygosity, the inclusion of both the pedigree heterosis coefficient and a genomic-based heterozygosity measure in the analysis of crossbred cattle populations could be beneficial to limiting prediction bias for some traits. The benefits associated with the accurate prediction of the carcass merit an animal will achieve at slaughter have been previously discussed in detail (Kenny et al., 2020a; Kenny et al., 2020b). The benefits, to the Irish beef industry at least, revolve around enabling the prescription of corrective measures in the production cycle of cattle that are predicted to achieve carcass metrics that fail to align with the desires of the supply chain. The fact that both the pedigree-based heterosis coefficient and a genomic heterozygosity measure were both associated with CC and CF signifies that the two measures capture, to some extent, different aspects of heterosis effects. The heterosis coefficient represents a proxy for expected heterozygosity (Dickerson, 1973), while OH and HL reflects the extent to which an animal is heterozygous across its genome (at least based on the SNP included in the analysis), and ROHet reflects the presence of stretches of heterozygous SNPs in the genome. Given the fact there was little change in the model solution for the heterosis coefficient when ROHet was also included in the model, compared to the reduction in the corresponding model solution when OH and HL were included, signifies that the measure are distantly different. This is corroborated by the weak correlation between the heterosis coefficient and ROHet, as well as by their respective definitions.

### Single Nucleotide Polymorphism-Phenotype Dominance Associations

Akanno et al. (2018) in their genome-wide association analysis of CW, which included 6,794 cattle and genotype information from the Illumina Bovine SNP50 panel, failed to detect any SNPs that demonstrated a dominance association with CW; the current study found only a single SNP on BTA 5 demonstrating such an association with CW. No previous cattle study has attempted to locate dominance effects across the genome associated with either CC or CF. The dataset used for the association analyses in the present study comprised a relatively large, heterogeneous group of cattle that included both purebred animals, as well as animals that differed in crossbred combinations of 12 distinct breeds; ensuring the association analyses had sufficient power to identify SNPs demonstrating dominance associations should they truly exist. The largest dominance effect associated with each trait were 1.89 kg, −0.113 and 0.151 units for CW, CC (scored 1–15) and CF (scored 1–15) respectively. Furthermore, the sign of the dominance effects of each lead SNP was generally in agreement with that of the corresponding regression coefficients from the regression of the carcass traits on the heterosis coefficient or on the genomic heterozygosity measures. Nonetheless, the effects estimated for individual SNPs in the present study could, in some instances, be overestimated, which could be a reflection of the presence of linkage disequilibrium between SNPs associated with the trait in question. On the other hand, overestimation of SNP effects can be a common feature of genome-wide association analyses that use single-SNP models (Li and Kim, 2015). Moreover, genes located within 0.5 Mb of the QTL regions detected in the present study, such as MSTN, have been previously reported to contribute to phenotypic differences in the carcass traits of interest (Purfield et al., 2019). In addition, many SNPs detected to have associations with the traits of interest to the present study, namely those detected to have additive associations with CC and, to a lesser extent, additive associations with CF, were not present within clear peaks or signals. This has been previously attributed to numerous factors such as allele frequency, linkage disequilibrium and population structure, among others factors (Tabangin et al., 2009; Platt et al., 2010; Sesia et al., 2021). Finally, there was some overlap between the detected additive and dominance QTL regions in the present study; the presence of overlapping additive and dominance QTL regions have been detected in previous cattle studies (Kim et al., 2003; Powell et al., 2013; Li et al., 2017). In terms of interpretation, Powell et al. (2013) attributed the presence of dominance QTL regions that did not overlap with additive QTL regions to over-dominance, and those that overlapped with additive regions to dominance. In the absence of an additive association at a locus, it would be expected that there is no (significant) difference between the effects associated with the two homozygous genotypes for the trait in question; the lack of an additive association also signifies there is little to no difference between the effects of the two homozygous genotypes and their mean. The presence of a dominance association at the same locus signifies that the effect associated with the heterozygous genotype for the trait in question is significant greatly (or lesser) than the mean of the effects associated with the homozygotes. Therefore, the presence of a dominance association and lack of an additive association at a given locus should signify the presence of over-dominant (or under-dominant) expression. Furthermore, based on the definitions provided for additive and dominance SNP effects by Wolf et al. (2008), the presence of additive and dominance association at the same loci could reflect dominance expression, if not over- or under-dominant expression. With regard to genetic selection in populations of genotyped sires and dams, Dekkers and Chakraborty (2004) highlighted that the increase in response to selection, when that selection considered QTL regions that comprised both additive and dominance associations, was marginal. On the other hand, Dekkers and Chakraborty (2004) outlined a substantial increase in response to selection in breeding programs that consider QTL regions comprising loci associated with over- or under-dominant expression.

Knowledge of SNPs demonstrating dominance associations with traits of interest could facilitate the calculation of trait-specific genomic heterozygosity measures, which, in turn, could be useful for accurate phenotypic predictions. The calculation of such trait-specific measures could be based on SNPs from regions in which SNP-phenotype dominance associations have been detected for the trait in question. As a follow up analysis, trait-specific genomic heterozygosity measures were separately calculated using either 1) all SNPs from chromosomes on which a dominance effect was detected, or 2) all SNPs from chromosomes on which no dominance effect was detected. Both genomic heterozygosity measures were simultaneously included in the statistical model as fixed effects; each pair of measures was specific for each carcass trait, which was fitted as the dependent variable. For all carcass traits, only the heterozygosity measure derived from SNPs residing on chromosomes where a dominance effect was located was significant. On this basis, the definition of, and adjustment for, trait-specific heterozygosity measures, as opposed to global heterozygosity measures could be beneficial to the accurate prediction of phenotypic performance. Nonetheless, should trait-specific measures be used, an important consideration should be the power of the association analyses used to detect the dominance associations that underline the definition of such measures.

## Conclusion

The pedigree-based heterosis coefficient and the investigated measures of genomic heterozygosity, for the most part, independently contribute to the observed variation in carcass merit of crossbred cattle. This signifies that consideration should be given to the inclusion of both pedigree- and genomic-based heterozygosity measures in the analysis of crossbred cattle populations, at least for CC and CF. Additionally, the results of the present study demonstrated that there are relatively few SNPs which demonstrate dominance associations with the carcass traits of interest. The dataset used to detect these associations comprised high-density genotype data for a relatively large, heterogeneous group of crossbred cattle, with varying levels of heterozygous in their genomes. Therefore, the association analysis conducted should have sufficient power to detect dominance SNP-phenotype association for the carcass traits of interest, should they exist.

## Data Availability

The data analyzed in this study is subject to the following licenses/restrictions: Individual genotype and phenotype data used in this study are managed by a third party, the Irish Cattle Breeding Federation (ICBF). Reasonable requests for data can be made to the Irish Cattle Breeding Federation, Highfield House, Shinagh, Bandon, Co. Cork, Ireland (email: query@icbf.com; website: https://www.icbf.com/). All significant associations identified in the present study are provided within the article. Requests to access these datasets should be directed to Irish Cattle Breeding Federation, Highfield House, Shinagh, Bandon, Co. Cork, Ireland; email: query@icbf.com; website: https://www.icbf.com.

## Abbreviations

BTA, Bos tarsus chromosome number; CC, carcass conformation; CF, carcass fat; CW, carcass weight; HL, homozygosity by locus; OH, observed heterozygosity; ROHet, runs of heterozygosity; SNPs, single nucleotide polymorphisms.
