# Supplementary material for: The Association Between Genomic Heterozygosity and Carcass Merit in Cattle
Source: Front Genet. 2022 Feb 24;13:789270. doi: 10.3389/fgene.2022.789270 (PMC8908906; doi:10.3389/fgene.2022.789270)
Supplement: Supplementary file 1 [file DataSheet1.docx]

**Supplementary material**

**Title: The association between genomic heterozygosity and carcass merit in cattle**

**Authors:** David Kenny, Tara R. Carthy, Craig P. Murphy, Roy D. Sleator, Ross D. Evans and Donagh P. Berry

**Supplementary Table S1** Mean, standard deviation, minimum and maximum for the heterosis coefficient and for genomic heterozygosity measures, namely observed heterozygosity and homozygosity by locus and runs of heterozygosity. Summary statistics included for runs of heterozygosity include those for the number of runs and the length of the runs

|  | Mean | Standard deviation | Minimum | Maximum |
| --- | --- | --- | --- | --- |
| Heterosis | 0.69 | 0.34 | 0 | 1 |
| Observed heterozygosity | 0.37 | 0.02 | 0.23 | 0.43 |
| Homozygosity by locus | 0.41 | 0.02 | 0.26 | 0.45 |
| Number of runs of heterozygosity | 30 | 5.8 | 8 | 61 |
| Length of runs of heterozygosity (Kb) | 159.1 | 80.1 | 16.7 | 1323.5 |

**Supplementary Table S2** Akaike’s Information Criterion (AIC) values from the statistical models that include various combinations of the heterozygosity measures that included the heterosis coefficient (Het), observed heterozygosity (OH), homozygosity by locus (HL) and runs of heterozygosity.

| Heterosis/Heterozygosity measure(s) included in the model | AIC |
| --- | --- |
| Carcass weight (kg) |  |
| Het | 190829.72 |
| OH | 190804.05 |
| HL | 190790.03 |
| ROHet | 190851.44 |
| Het & OH | 190804.06 |
| Het & HL | 190790.41 |
| Het & ROHet | 190836.44 |
| Het, OH & ROHet | 190808.58 |
| Het, HL & ROHet | 190794.48 |
| Carcass conformation (scored 1 [poor] to15 [excellent]) |  |
| Het | 32986.77 |
| OH | 32982.41 |
| HL | 33010.54 |
| ROHet | 33034.85 |
| Het & OH | 32973.15 |
| Het & HL | 32990.49 |
| Het & ROHet | 33005.45 |
| Het, OH & ROHet | 32997.37 |
| Het, HL & ROHet | 32995.70 |
| Carcass fat (scored 1 [thin] to 15 [fat]) |  |
| Het | 53909.76 |
| OH | 53706.84 |
| HL | 53766.16 |
| ROHet | 54098.89 |
| Het & OH | 53687.83 |
| Het & HL | 53738.36 |
| Het & ROHet | 53898.90 |
| Het, OH & ROHet | 53697.77 |
| Het, HL & ROHet | 53746.04 |
